# Species-Specific miRNAs Contribute to the Divergence between Deciduous and Evergreen Species in *Ilex*

**DOI:** 10.3390/plants13111429

**Published:** 2024-05-21

**Authors:** Zhonglong Guo, Zhenxiu Xu, Lei Li, Ke-Wang Xu

**Affiliations:** 1 Co-Innovation Center for Sustainable Forestry in Southern China, College of Life Sciences, Nanjing Forestry University, Nanjing 210037, China; guozhl@njfu.edu.cn (Z.G.); xu123xzx@njfu.edu.cn (Z.X.); 2 State Key Laboratory of Protein and Plant Gene Research, School of Advanced Agricultural Sciences, Peking University, Beijing 100871, China

**Keywords:** miRNA, species specific, leaf abscission, *Ilex*

## Abstract

MicroRNAs (miRNAs) are pivotal regulators of gene expression, playing crucial roles in plant developmental processes and environmental responses. However, the function of miRNAs in influencing deciduous traits has been little explored. Here, we utilized sRNA-seq on two deciduous species, *Ilex polyneura* (Hand.-Mazz.) S. Y. Hu and *Ilex asprella* Champ. ex Benth., along with an evergreen species, *Ilex latifolia* Thunb., to identify and annotate miRNAs within these species. Our analysis revealed 162 species-specific miRNAs (termed SS-miRNAs) from 120 families, underscoring the fundamental roles and potential influence of SS-miRNAs on plant phenotypic diversity and adaptation. Notably, three SS-miRNAs in *I. latifolia* were found to target crucial genes within the abscission signaling pathway. Analysis of *cis*-regulatory elements suggested a novel regulatory relationship that may contribute to the evergreen phenotype of *I. latifolia* by modulating the abscission process in a light-independent manner. These findings propose a potential mechanism by which SS-miRNAs can influence the conserved abscission pathway, contributing to the phenotypic divergence between deciduous and evergreen species within the genus *Ilex*.

## 1. Introduction

MicroRNAs are pivotal non-coding RNA molecules that regulate gene expression across eukaryotes [1]. These small 20–24 nucleotide miRNAs derive from longer hairpins and bind to complementary sites on target transcripts, leading to mRNA degradation or translational repression [2,3,4]. In plants, extensive studies have established the regulatory functions of miRNAs in development, reproduction, environmental responses, and interspecies communication [3,5,6,7,8]. Notably, functionally crucial miRNAs undergo rapid evolution, exhibiting a wealth of lineage-specific or evolutionarily young miRNAs [9,10,11]. A comprehensive analysis across 81 plant species, phylogenetically ranging from chlorophytes to angiosperms, revealed that over 61.2% of miRNA families were only presented in one species (termed species-specific miRNAs, or SS-miRNAs), which were implicated in a variety of biological functions and metabolic processes [9]. For instance, the SS-miRNA in *Arabidopsis thaliana*, miR775, has been shown to regulate leaf size and cell wall pectin levels by targeting *GALT9* [12]. These studies underscore the significance of exploring the evolutionary and biological roles of SS-miRNAs, contributing to the understanding of plant diversity and adaptation.

Leaf abscission, the process by which plants shed their leaves, involves a complex molecular mechanism that differs between deciduous and evergreen plants [13,14]. In deciduous species, leaves fall off as the seasons change, primarily during autumn and winter. Environmental cues, such as changes in photoperiod, often trigger leaf abscission, leading to the activation of genes related to cell wall degradation in the abscission zone [14,15]. Evergreen species, as the name suggests, remain green throughout the year. Unlike deciduous species, evergreens do not shed their leaves in response to seasonal changes, exhibiting a more random pattern of leaf abscission [16]. At present, numerous studies have focused on the mechanisms regulating leaf shedding and the activation of abscission signals [11,12,13,14,15]. Within this regulatory signaling pathway, *HAE/HSL2*, as the receptors located on the cell membrane bind to IDA ligands, transmitting signals to the downstream mitogen-activated protein kinase (MAPK) cascade, through unknown means [17,18,19,20]. Agamous-like 15 (AGL15), a MADS domain transcriptional factor, represses the transcription of *HAE/HSL2* by binding to their promoters [21]. Upon the activation of abscission, the MAPK cascade phosphorylates AGL15, then de-represses *HAE/HSL2* expression [20,22]. Consequently, *HAE/HSL2* can be transcribed and transported to the cell membrane, forming a positive feedback loop of the abscission signal pathway [14]. Proper abscission also requires other genes, although it is not entirely clear how these fit into the aforementioned positive feedback loop. For instance, EVERSHED (EVR), a leucine-rich repeat receptor-like kinase, functions as an inhibitor of abscission [23]. By comparison, our understanding of the distinction in the abscission signaling pathway between deciduous and evergreen plants remains relatively poor.

*Ilex* L., the sole and species-rich genus in the Aquifoliaceae family, also known as the holly family, comprises more than 600 deciduous or evergreen trees or shrubs [24,25,26].

The dominant species in holly are broad-leaved species, which are an important component of the tropical and subtropical forest ecosystems and are commonly used as ornamental plants in parks and gardens [27,28,29]. Recently, genome and transcriptome sequencing in holly have been conducted for unraveling the genetic structure and understanding the biological principles of the diverse traits [30,31,32,33]. However, the systematic identification and annotation of miRNAs in holly are only sketchy.

In this study, we performed small RNA sequencing (sRNA-seq) on two deciduous species, *Ilex polyneura* (Hand.-Mazz.) S. Y. Hu and *Ilex asprella* Champ. ex Benth., as well as on one evergreen species, *Ilex latifolia* Thunb. Through comprehensive bioinformatic analysis, we identified 171 miRNAs in *I. asprella*, 184 miRNAs in *I. polyneura*, and 177 miRNAs in *I. latifolia*, establishing the first miRNA portfolio for the Aquifoliaceae family. A comparative analysis with known miRNAs in land plants revealed that 30.5% of these miRNAs are species-specific. We further delineated a model mediated by SS-miRNAs for regulating the abscission activation signaling pathway. We showed that three SS-miRNAs in the evergreen species *I. latifolia* regulated target genes associated with leaf abscission. *Cis*-regulatory elements of promoters indicated that these three SS-miRNAs were not responsive to light, despite the presence of multiple light responsive elements on the promoters of their target genes. Our findings suggest a novel mechanism by which SS-miRNAs in *I. latifolia* may enable the abscission pathway to bypass light responsiveness, potentially contributing to its evergreen nature.

## 2. Results

### 2.1. Identification of miRNAs in Hollies

**Figure 1:**
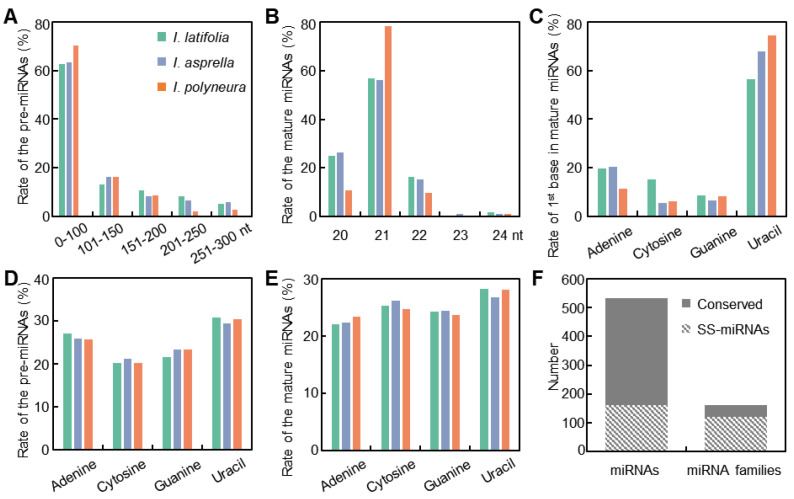
Identification and annotation of miRNAs in three species of *Ilex*. (**A**,**B**) Length of pre-miRNAs (**A**) and mature miRNAs (**B**), respectively. (**C**) Base composition of the first nucleotide at the 5′ end of mature miRNAs. (**D**,**E**) Base composition of pre-miRNAs (**D**) and mature miRNAs (**E**), respectively. (**F**) Number of conserved and SS-miRNAs in the three species of *Ilex*, alongside their corresponding miRNA families.

To comprehensively identify miRNAs in holly, over 204 million sRNA reads were generated from branches with leaves of *I. asprella*, *I. polyneura*, and *I. latifolia* (details in Section 4). Subsequently, a classical pipeline adhering to the newly updated criteria, miRDeep-P2, was implemented to detect miRNAs [34,35]. Finally, 171 miRNAs in *I. asprella*, 184 miRNAs in *I. polyneura*, and 177 miRNAs in *I. latifolia* were identified (Tables S1–S3). Analysis of the intrinsic characteristics of the miRNAs revealed that the majority of precursor sequences were shorter than 100 nucleotides (Figure 1A), the typical length of mature miRNAs was 21 nucleotides (Figure 1B), and the initial nucleotide of mature miRNAs was predominantly uracil (Figure 1C). The base composition of pre-miRNAs predominantly consisted of adenine and uracil (Figure 1D), while adenine was relatively less abundant in mature miRNAs (Figure 1E). Using over 38 thousand known miRNAs in PmiREN as a standard, all miRNAs in hollies were further annotated into 162 families using the homology method [36,37]. Upon cross-validation among the three species of hollies, 162 miRNAs (30.5%) from 120 families (74.1%) were identified as species-specific or novel, indicating that most SS-miRNA families contain low copies (Figure 1F).

### 2.2. Characteristic of SS-miRNAs in Hollies

**Figure 2:**
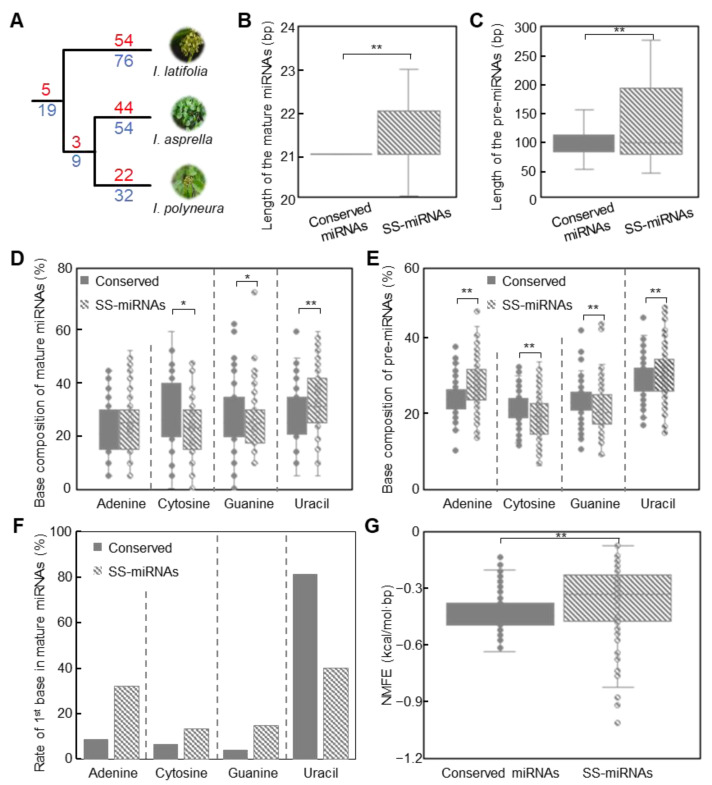
Characteristics of conserved miRNAs and SS-miRNAs in *Ilex*. (**A**) Origin of SS-miRNAs in *Ilex*. The number of miRNA families is marked above the lines (red) and the number of miRNAs is marked below the lines (blue). (**B**,**C**) Comparison of length between conserved miRNAs and SS-miRNAs in mature miRNAs (**B**) and pre-miRNAs (**C**). (**D**–**F**) Comparison of base composition between conserved miRNAs and SS-miRNAs in mature miRNAs (**D**), pre-miRNAs (**E**), and the first base in mature miRNAs (**F**). (**G**) NMFE of pre-miRNAs between conserved miRNAs and SS-miRNAs. The solid rectangles indicate conserved miRNAs, while dashed rectangles represent SS-miRNAs. * *p* < 0.05 and ** *p* < 0.01 using an independent samples *t*-test.

Further analysis focusing on the evolutionary conservation of miRNAs revealed a conserved set of nineteen miRNAs from five families present in all three *Ilex* species, contrasting with their absence in other species of land plants (Figure 2A). Additionally, a subset of nine miRNAs across three families was common to *I. asprella* and *I. polyneura*, but was not detected in *I. latifolia* (Figure 2A). Investigation into the species specificity of miRNAs uncovered that *I. latifolia* contained 76 SS-miRNAs from 54 families, *I. asprella* had 54 SS-miRNAs from 44 families, and *I. polyneura* possessed 32 SS-miRNAs from 22 families (Figure 2A).

Then, we conducted a comparative analysis of sequence characteristics between conserved miRNAs and SS-miRNAs. Our results suggested that SS-miRNAs, in both mature and precursor sequences, exhibited significantly greater lengths than those of conserved miRNAs (Figure 2B,C). Analysis for the length of mature sequences highlighted the dominance of 21 nucleotides in conserved miRNAs, while SS-miRNAs were slightly longer, averaging about 21.4 nucleotides (Figure 2B). The average length of precursor sequences of SS-miRNAs is 126.6 nucleotides, significantly longer than that of conserved miRNAs, which average 100.5 nucleotides (Figure 2C). In terms of base composition, the mature sequences of SS-miRNAs revealed a significantly higher uracil content relative to conserved miRNAs (Figure 2D). The precursor sequences also showed marked differences in base composition between the two sets of miRNAs, with SS-miRNAs displaying elevated levels of uracil and adenine (Figure 2E). The bias toward uracil as the initial base at the 5′ end of mature sequences was a common characteristic in both SS-miRNAs and conserved miRNAs (Figure 2F). Notably, our results also found that the skew to adenine in SS-miRNAs was significantly higher than that in conserved miRNAs (Figure 2F). The Normalized Minimum Free Energy (NMFE), which measures the stability of the secondary structures of precursor miRNAs, showed a significant distinction between SS-miRNAs and conserved miRNAs, suggesting more stable structures of conserved miRNAs compared to those of SS-miRNAs (Figure 2G).

### 2.3. Function of SS-miRNAs in I. latifolia

*Ilex latifolia* has the highest number of SS-miRNAs among the three species of *Ilex* and displays significantly unique traits compared to the other two species, such as deciduousness, plant height, and the color of flower and fruit. Therefore, this study specifically concentrates on investigating the biological function of SS-miRNAs in *I. latifolia* in the following analysis.

Using an in silico approach, we identified 5665 putative target genes for 101 conserved miRNAs and 6026 target genes for 76 SS-miRNAs, indicating that all miRNAs in *I. latifolia* potentially target specific protein-coding genes. Consistent with the previous hypothesis that novel miRNAs possess more target genes that appear at random in the genome [38], we observed that SS-miRNAs regulated more target genes than conserved miRNAs.

**Figure 3:**
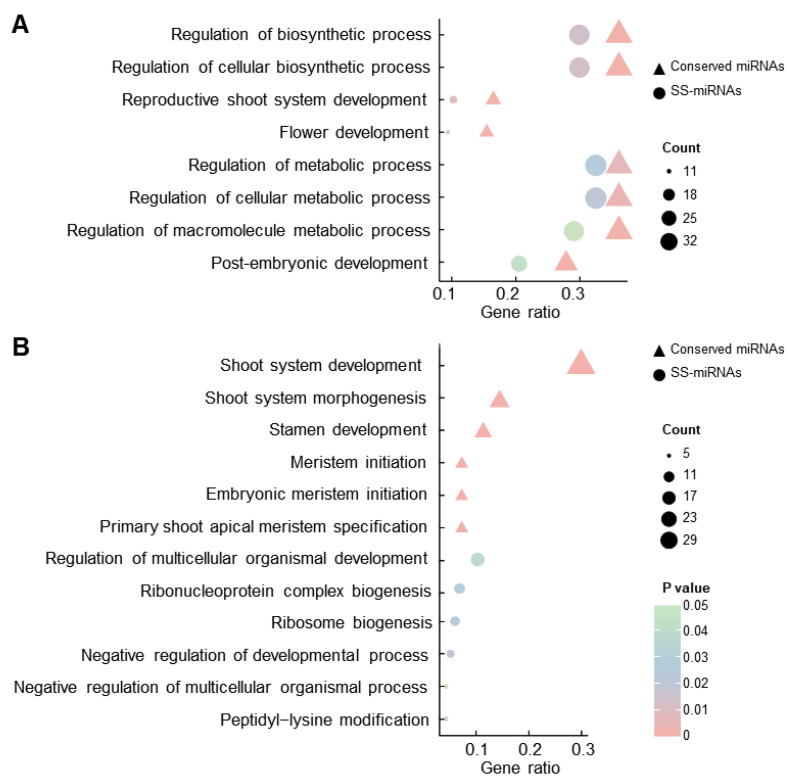
GO enrichment for the target genes of conserved miRNAs and SS-miRNAs in *I. latifolia*. (**A**) GO enrichment analysis showing the common terms for the target genes of conserved miRNAs and SS-miRNAs. (**B**) The top six unique GO terms for the target genes of conserved miRNAs and SS-miRNAs. The vertical axis indicates the descriptions of the GO terms. The color and size of the dots symbolize the *p* value and the count of target genes associated with each term, respectively.

Gene Ontology (GO) analysis revealed that target genes of both conserved miRNAs and SS-miRNAs are commonly enriched in eight terms associated with various biosynthetic and metabolic processes, as well as tissue development (Figure 3A). Notably, conserved miRNAs exhibited a higher gene ratio and lower *p* value compared to SS-miRNAs (Figure 3A). Additionally, our results highlighted that several GO terms were uniquely enriched for the target genes of conserved miRNAs and SS-miRNAs. The top six terms for the function of conserved miRNAs related to the development and morphogenesis of shoot, stamen development, as well as the initiation of multiple meristems (Figure 3B). In contrast, the functions of SS-miRNAs are enriched in the GO terms involved in multicellular organismal development, ribosome-related biogenesis.

### 2.4. SS-miRNAs Related to Leaf Abscission

**Figure 4:**
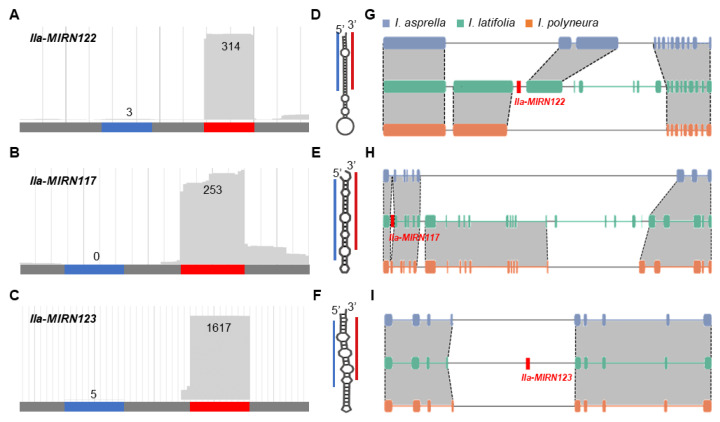
Three SS-miRNAs related to leaf abscission in *I. latifolia*. (**A**–**C**) Reads distribution along the precursor sequences of *Ila-MIRN122* (**A**), *Ila-MIRN117* (**B**), and *Ila-MIRN123* (**C**). The number show the reads mapped to mature and star miRNA. (**D**–**F**) Secondary structures of *Ila-MIRN122* (**D**), *Ila-MIRN117* (**E**), and *Ila-MIRN123* (**F**) are predicted using RNAfold [39]. Red lines indicate the positions of mature miRNAs, while blue lines show the positions of star miRNAs. (**G**–**I**) Synteny analysis shows *Ila-MIRN122* (**G**), *Ila-MIRN117* (**H**), *Ila-MIRN123* (**I**), and their respective adjacent genes in *I. latifolia* against syntenic blocks in *I. asprella* and *I. polyneura*. Exons in *I. asprella*, *I. latifolia*, and *I. polyneura* are marked in blue, green, and orange, respectively.

The results of the GO enrichment of SS-miRNAs in *I. latifolia* were unexpected. Therefore, we delved further into the detailed developmental processes that SS-miRNAs were involved in. We identified three SS-miRNAs in *I. latifolia* that were implicated in regulating genes associated with leaf abscission. We examined the distribution of sRNA-seq reads across the precursor sequences of these miRNAs (*MIRN122*, *MIRN117*, and *MIRN123*). The findings indicated a canonical distribution pattern for miRNAs, where most reads were concentrated in the mature miRNA sequences, with fewer reads in the star sequences (the complementary strand-forming part of the miRNA:miRNA* duplex) (Figure 4A–C and Figure S1). This pattern, along with the predicted secondary structures of the SS-miRNAs (Figure 4D–F), suggested that these miRNAs were bona fide miRNAs. Additionally, a syntenic analysis of the three SS-miRNAs with other *Ilex* species (*I. asprella* and *I. polyneura*) revealed no syntenic blocks, indicating these miRNAs had originated recently and were unique to *I. latifolia* (Figure 4G–I). Thus, we detected three SS-miRNAs in *I. latifolia* that potentially regulate the target genes involved in leaf abscission.

**Figure 5:**
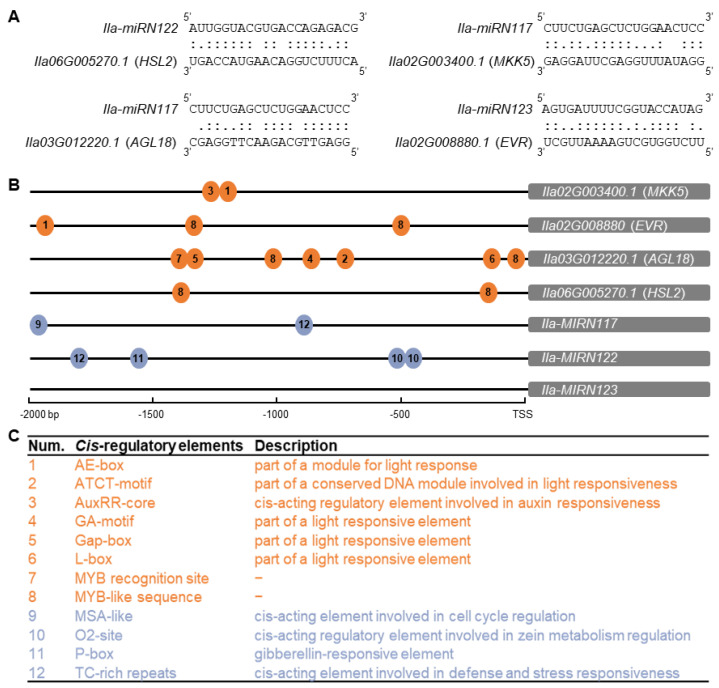
*Cis*-regulatory element analysis reveals distinct responses to light between the conserved genes and SS-miRNAs involved in the abscission pathway. (**A**) Three SS-miRNAs in *I. latifolia* regulate four target genes, *HSL2*, *MKK5*, *ALG18*, and *EVR*, involved in the signaling pathway of organ abscission. (**B**,**C**) *Cis*-regulatory element analysis on the promoters of three SS-miRNAs and their target genes (**B**) and the corresponding description of each element (**C**). The *cis*-regulatory elements on the four target genes and the three SS-miRNAs are marked in orange and blue, respectively.

**Figure 6:**
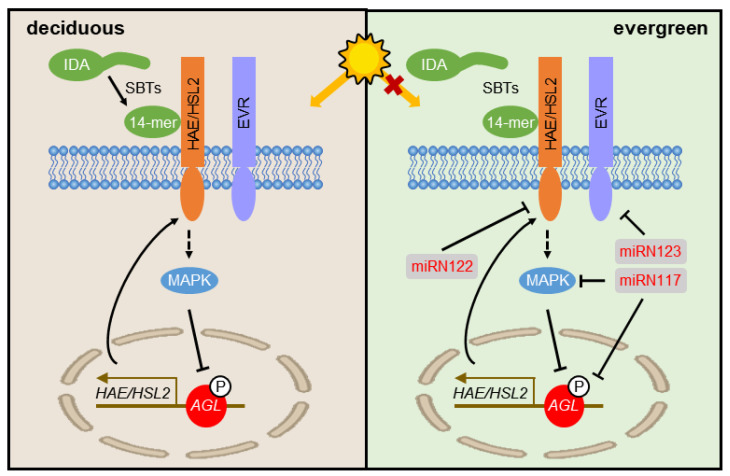
Model of the abscission activation signaling pathway in deciduous and evergreen hollies. In two deciduous hollies, *I. asprella* and *I. polyneura*, a classical abscission activation signaling pathway is identified. The 14-mer peptide, resulting from SBTs cleaving IDA, binds to both HAE/HSL2 and its co-receptor. Then, HAE/HSL2 initiates an MAPK cascade through an unidentified intermediate. Following the phosphorylation of AGL by MAPK, *HAE/HSL2* transcription is de-repressed. A positive feedback loop is subsequently completed when newly generated HAE/HSL2 is transported to the plasma membrane. The core genes of this pathway are regulated by light. In *I. latifolia*, an evergreen species of *Ilex*, three SS-miRNAs fine-tune the expression of core genes, escaping from the light responsiveness.

Bioinformatic analysis revealed that three SS-miRNAs in *I. latifolia* target the exons of four genes critical to leaf abscission (Figure 5A). Ila-miRN122 targets *HSL2*, a receptor-like protein kinase that plays a crucial role in abscission activation (Figure 5A). Ila-miRN117 targets *MKK5*, situated downstream of the HAE/HSL2 receptor complex, amplifying the abscission signal (Figure 5A). Additionally, Ila-miRN117 targets *AGL18*, the *Arabidopsis* homolog of *AGL15*, acting as a negative regulator of abscission (Figure 5A). The transcriptional factor AGL15, once phosphorylated by the MAPK cascade, may de-repress the expression of *HAE*. Ila-miRN123 targets the homolog of *EVR*, a leucine-rich repeat receptor-like kinase, functioning as an inhibitor of leaf abscission (Figure 5A). An analysis of the *cis*-regulatory elements on the promoters of SS-miRNAs and their target genes showed that the promoters of conserved target genes contain a variety of light-responsive elements (Figure 5B,C). In contrast, the promoters of the three SS-miRNAs lacked light-responsive elements (Figure 5B,C). These findings imply that while the conserved genes in the abscission pathway are responsive to light, the SS-miRNAs, which serve as the novel regulators within this pathway, do not respond to light (Figure 6).

## 3. Discussion

Genetic innovations enable plants to develop adaptive traits for diverse environmental conditions [40,41,42]. miRNAs, a crucial class of endogenous regulatory RNAs, regulate a variety of life processes in plants [3,6]. Numerous studies have indicated that SS-miRNAs, as new regulators of the gene regulatory network, are involved in the unique adaptations of different species [9,43,44]. In our study, we conducted a comprehensive investigation of miRNAs within three *Ilex* species. We analyzed the characteristics of these miRNAs, including the lengths of pre-miRNAs and mature miRNAs (Figure 1A,B), as well as the base composition of the first nucleotide at the 5′ end of mature miRNAs (Figure 1C), pre-miRNAs (Figure 1D), and mature miRNAs (Figure 1E). Our findings corroborate the characteristics of plant miRNAs described in prior studies [45], underscoring the reliability of miRNA identification and annotation in this study. Cross-validation revealed 162 SS-miRNAs, constituting 30.5% of those examined from 120 families (Figure 1F). This study is the first to document SS-miRNAs within the Aquifoliaceae family, although the proportion of SS-miRNAs is relatively lower compared to those found in other angiosperm families [9]. A comparative analysis of the characteristics between conserved miRNAs and SS-miRNAs exhibits significant differences (Figure 2), suggesting a potential evolutionary trajectory for SS-miRNAs towards acquiring canonical features akin to those found in conserved miRNAs [46]. Functional analysis revealed that SS-miRNAs were primarily involved in regulating various metabolic processes (Figure 3A), consistent with findings from earlier studies [9,47]. Furthermore, our investigation highlighted the function of SS-miRNAs in the development of multicellular organisms and ribosome-related biogenesis, which was uncommon in previous studies (Figure 3B).

The trait of deciduousness, by which plants shed their leaves seasonally, represents a complex evolutionary characteristic among angiosperms [48,49,50]. These plants exhibit a broad spectrum of leaf phenologies, including deciduous, evergreen, and semi-evergreen habits, each adapted to specific environmental conditions. The shift between deciduous and evergreen features has occurred multiple times independently across various angiosperm lineages. Yet, the molecular mechanisms behind these frequent transitions remain elusive. In our study, we identified three SS-miRNAs in the evergreen species *I. latifolia* that regulated four target genes involved in leaf abscission (Figure 4). However, in the deciduous relatives, *I. asprella* and *I. polyneura*, we did not find any miRNA targeting genes in the abscission pathway. Our analysis of *cis*-regulatory elements suggested that, while the conserved genes in the abscission pathway are responsive to light, the SS-miRNAs are not (Figure 5).

In this study, we propose an SS-miRNA mediated model for the abscission activation signaling pathway between deciduous and evergreen hollies (Figure 6). In the deciduous hollies, *I. asprella* and *I. polyneura*, a classical abscission activation signaling pathway is identified, where a 14-mer peptide, resulting from SBTs cleaving IDA, binds both HAE/HLS2 and its co-receptor. Then, HAE initiates an MAPK cascade through an unidentified intermediate. Following the phosphorylation of AGL by MAPK, *HAE* transcription is de-repressed. A positive feedback loop is subsequently completed when newly generated HAE is transported to the plasma membrane. The core genes of this pathway are regulated by light. In *I. latifolia*, an evergreen species of *Ilex*, three SS-miRNAs regulate the expression of core genes, thereby escaping from the responsiveness to light. Given the frequent transitions between deciduous and evergreen traits within angiosperms, further research in other lineages is needed to explore this mechanism, by which producing novel miRNAs fine-tunes the conserved abscission pathway, resulting in changes in deciduousness.

The regulation of abscission is a complex process affected by photoperiod and other factors, such as temperature, stress, and hormones [14,15,51,52]. High levels of auxin in the cells of the abscission zone prevent the initiation of abscission [53]. In contrast, ethylene triggers this process, resembling the natural increase in ethylene production associated with organ aging, fruit ripening, or response to herbivore damage [54,55,56]. This study sheds light on the potential role of SS-miRNAs in bypassing the influence of light. The mechanisms behind leaf abscission in response to external factors in deciduous versus evergreen species require further investigation.

## 4. Materials and Methods

### 4.1. Plant Materials

The plant materials used for sequencing in this study were collected from fresh leaf tissues of *I. latifolia*, *I. polyneura*, and *I. asprella*. The *I. latifolia* and *I. asprella* plants are growing on the campus of Nanjing Forestry University, while the *I. polyneura* plant is growing in the Kunming Botanical Garden. Approximately 500 mg of leaf tissue was dissected and stored in liquid nitrogen until it was delivered in dry ice; then, these samples were used for sRNA-seq. The phylogenetic relationship of three species in this study is conducted according to previous studies [26,57].

### 4.2. Small RNA Libraries Construction and Sequencing

Total RNA from *I. latifolia*, *I. polyneura*, and *I. asprella* was isolated from leaves using Trizol reagent (Invitrogen, Waltham, MA, USA). The small RNA was isolated following the protocol from BGI (Extraction of Plant RNA BGI-NGS-TQ-RNA-005 A0). Small RNA cDNA libraries were prepared using the MGIEasy Small RNA Library Prep Kit (MGI Tech Co., Ltd., Shenzhen, China) based on the user manual. Briefly, a specific quantity of RNA sample is first purified and then mixed with a 3′ adapter, followed by incubation. Next, a 5′ adapter is added and the mixture is incubated again. The Reverse Transcription Reaction Mixture is prepared and subjected to a thermocycler. The program is set to incubate for 60 min at 50 °C, followed by 15 min at 70 °C, and is then maintained at 4 °C. Similarly, the PCR Reaction Mixture is prepared and placed in the thermocycler, which runs the following program: 1 cycle at 95 °C for 3 min, followed by 15 cycles of 98 °C for 20 s, 56 °C for 15 s, and 72 °C for 15 s, concluding with an extension at 72 °C for 15 min and hold at 4 °C. The PCR products are then purified using PAGE gel and the recovered products are dissolved in EB solution. The choice of library quality control protocol depends on the specific requirements of the product. Single-stranded PCR products are generated through denaturation. The reaction setup and program for circularization are then established. This process results in single-stranded circularized products, while uncyclized linear DNA molecules are digested. Finally, the verified cDNA libraries undergo sequencing using MGISEQ-2000 (MGI Tech Co., Ltd., Shenzhen, China).

### 4.3. Identification of Conserved and SS-miRNAs

We perform a standardized process centered on miRDeep-P2 [35] and the latest plant miRNA criteria [45] to identify miRNAs from sRNA-seq datasets. For each species, whole genome reference and sRNA-Seq datasets served as input files of miRDeep-P2, with the new plant miRNA criteria applied as a filter. We utilized the comprehensive database PmiREN2.0 (Plant microRNA Encyclopedia) [36,37], which contains all identified miRNA sequences from various plants, to compare with the mature miRNA sequences of *I. latifolia*, *I. polyneura*, and *I. asprella*. By aligning these sequences with miRNAs from PmiREN2.0 using Blastn [58], we identified conserved miRNAs across the three species. Subsequently, by excluding these conserved miRNAs and re-comparing the remaining miRNA mature sequences with Blastn, we were able to identify miRNAs specific to the genus *Ilex* and those unique to each species (SS-miRNAs).

### 4.4. Prediction of Secondary Structures of Pre-miRNAs

The RNAfold WebServer (http://rna.tbi.univie.ac.at/cgi-bin/RNAWebSuite/RNAfold.cgi, accessed on 22 January 2024) is used to predict the potential secondary structures of pre-miRNAs. To utilize RNAfold, the precursor sequences of miRNAs are submitted using the default parameter settings.

### 4.5. Targets Prediction of Identified miRNAs

PsRNATarget (Schema V2, https://www.zhaolab.org/psRNATarget/analysis, accessed on 22 January 2024) is a bioinformatics tool designed for predicting miRNA target genes by evaluating the base complementary pairing between miRNA and mRNA. In this study, the miRNA target genes were identified by setting consistent PsRNATarget software parameters with previous studies [47,59,60]. The parameters are as follows: # of top targets is set to 200, expectation is set to 5, penalty for G:U pair is set to 0.5, penalty for other mismatches is set to 1, extra weight in seed region is set to 1.5, seed region is set to 2–13 nt, # of mismatches allowed in seed region is set to 2, HSP size is set to 19, penalty for opening gap is set to 2, penalty for extending gap is set to 0.5, and the translation inhibition range is set to 10–11 nt.

### 4.6. GO Analysis

Annotations of Gene Ontology (GO) terms were obtained from HollyGTD (https://hollygdb.com/, accessed on 22 January 2024) [31]. The associations of the predicted target genes of SS-miRNAs and conserved miRNAs with the GO terms were analyzed using custom scripts. Fisher’s exact test with the Benjamini–Hochberg correction was used to find enriched GO terms, with the adjusted *p* value set as 0.05.

### 4.7. Cis-Regulatory Element Analysis

The potential promoters of miRNAs and protein coding genes with 2000 bp were extracted using the in-house Perl script. Then, the sequences of each promoter were submitted to PlantCare (https://bioinformatics.psb.ugent.be/webtools/plantcare/html/, accessed on 22 January 2024) for predicting *cis*-regulatory elements [61]. The annotation of each element was also retrieved from the PlantCare database.

## Data Availability

The sequencing data for this study can be found in the NCBI Sequence Read Archive (SRA) under accession number PRJNA1092364.

## Supplementary Materials

The following supporting information can be downloaded at https://www.mdpi.com/article/10.3390/plants13111429/s1, Table S1: Summary of identified miRNAs in *I. asprella*; Table S2: Summary of identified miRNAs in *I. polyneura*; Table S3: Summary of identified miRNAs in *I. latifolia*. Figure S1: Reads distribution of three SS-miRNAs related to leaf abscission in *I. latifolia*.
