# Supplementary material for: Species-Specific miRNAs Contribute to the Divergence between Deciduous and Evergreen Species in Ilex
Source: Plants (Basel). 2024 May 21;13(11):1429. doi: 10.3390/plants13111429 (PMC11174832; doi:10.3390/plants13111429)
Supplement: Supplementary file 1 [file plants-13-01429-s001.zip › SFigure1_finial.pdf]

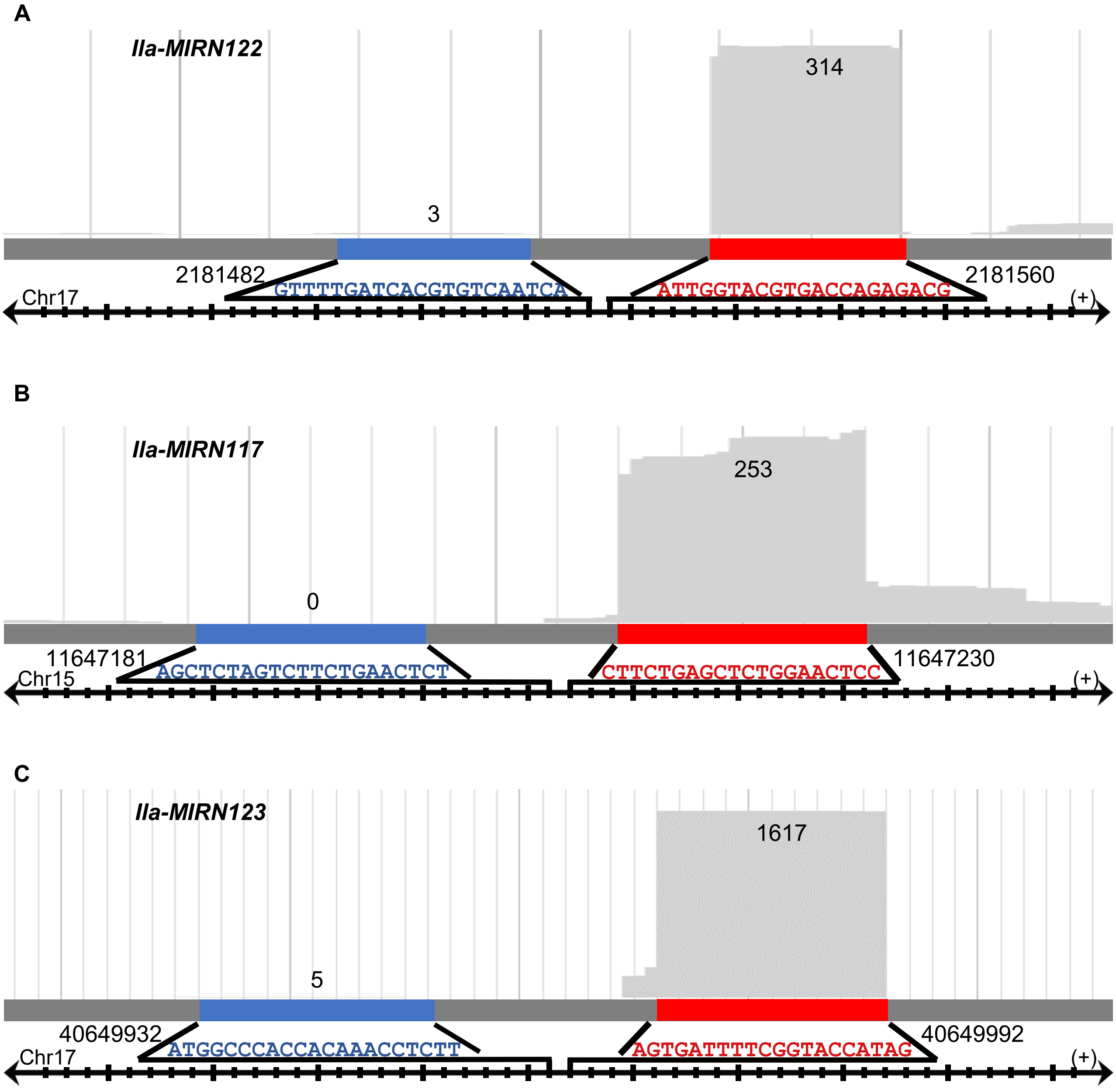

Figure S1. Reads distribution of three SS-miRNAs related to leaf abscission in *I. latifolia*. The number shows the reads mapped to mature and star miRNA. Letters with red and blue indicate the sequences of mature and star miRNAs, respectively.
